# HIPK2 as a Novel Regulator of Fibrosis

**DOI:** 10.3390/cancers15041059

**Published:** 2023-02-07

**Authors:** Alessia Garufi, Giuseppa Pistritto, Gabriella D’Orazi

**Affiliations:** 1 Unit of Cellular Networks, Department of Research and Advanced Technologies, IRCCS Regina Elena National Cancer Institute, 00144 Rome, Italy; 2 Centralized Procedures Office, Italian Medicines Agency (AIFA), 00187 Rome, Italy; 3 Department of Neurosciences, Imaging and Clinical Sciences, University “G. D’Annunzio”, 66013 Chieti, Italy

**Keywords:** HIPK2, fibrosis, kidney fibrosis, lung fibrosis, liver fibrosis, cardiac fibrosis, cancer-associated fibrosis, fibroblasts, myofibroblasts, cancer-associated fibroblasts (CAF)

## Abstract

**Simple Summary:**

Fibrosis can affect almost every organ and represents an increasing cause of morbidity and mortality worldwide. Despite significant progress in our understanding of the pathobiology of fibrosis, there is still a lack of putative anti-fibrotic targets to be exploited in anti-fibrosis therapies or used as biomarkers of fibrosis progression. The discovery that HIPK2 can control molecular pathways involved in fibrosis has opened a new field of study in both pathophysiology and the treatment of fibrosis.

**Abstract:**

Fibrosis is an unmet medical problem due to a lack of evident biomarkers to help develop efficient targeted therapies. Fibrosis can affect almost every organ and eventually induce organ failure. Homeodomain-interacting protein kinase 2 (HIPK2) is a protein kinase that controls several molecular pathways involved in cell death and development and it has been extensively studied, mainly in the cancer biology field. Recently, a role for HIPK2 has been highlighted in tissue fibrosis. Thus, HIPK2 regulates several pro-fibrotic pathways such as Wnt/β-catenin, TGF-β and Notch involved in renal, pulmonary, liver and cardiac fibrosis. These findings suggest a wider role for HIPK2 in tissue physiopathology and highlight HIPK2 as a promising target for therapeutic purposes in fibrosis. Here, we will summarize the recent studies showing the involvement of HIPK2 as a novel regulator of fibrosis.

## 1. Introduction

**Figure 1:**
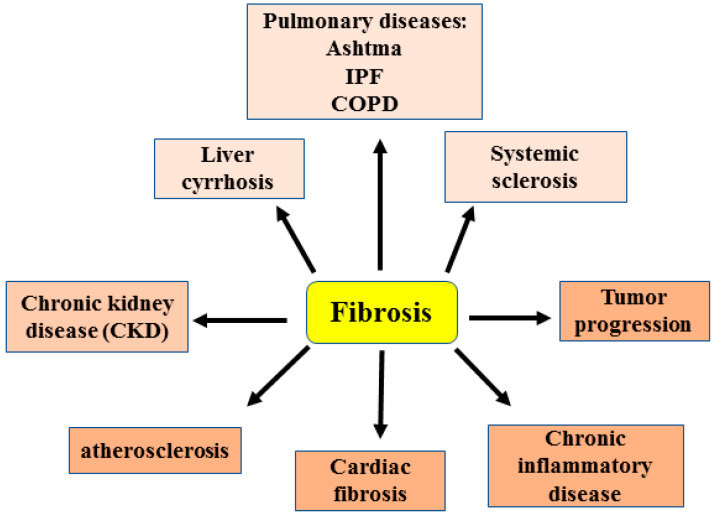
Schematic representation showing the involvement of fibrosis in several diseases (as explained in the text). IPF: idiopathic pulmonary fibrosis. COPD: chronic obstructive pulmonary disease.

The disproportionate accumulation of extracellular matrix (ECM) components in response to an injury, usually as a consequence of chronic inflammation, leads to fibrotic disorders that represent an increasing cause of morbidity and mortality worldwide. Fibrosis can affect nearly every organ and induce their dysfunction by disrupting the physiological tissue architecture, [1]. Fibrosis is directly and indirectly implicated in many common diseases including liver cirrhosis, asthma, chronic obstructive pulmonary disease (COPD), chronic kidney diseases (CKD), cardiac dysfunction, atherosclerosis, chronic inflammatory bowel diseases and idiopathic pulmonary fibrosis (IPF), or can manifest as systemic disease such as systemic sclerosis (SSc) [2,3]. In addition, fibrosis can contribute to cancer progression: the stiffened stroma indeed enhances tumor cell growth and promotes invasion and metastasis [4] (Figure 1). At the cellular level, activated fibroblasts are responsible for ECM deposition in both physiological conditions such as tissue repair and in pathological conditions such as prolonged tissue injury and chronic inflammation leading to fibrosis [5].

At the molecular level, the best characterized pro-fibrotic mediator is transforming growth factor (TGF)-β, a pluripotent growth factor that activates fibroblasts into myofibroblasts to stimulate the release of ECM proteins (e.g., collagens, elastins, proteoglycans, etc.) [6]. The Wnt/β-catenin pathway also plays an important role in pathological fibrosis [7]; excessive activation of fibroblasts can likewise be associated with the high expression of Notch1 and/or Notch3 genes [8].

In this review, we will first briefly summarize the cellular and molecular mechanisms of fibrosis that have been extensively described in many interesting reviews (for a review see refs. [1,3,9,10]). Then, the involvement of homeodomain-interacting protein kinase 2 (HIPK2) in several fibrotic diseases will be recapitulated. HIPK2 has been extensively studied for its role in restraining tumor progression [11]. However, since it can regulate pro-fibrotic pathways such as TGF-β, Wnt/β-catenin and Notch, it has also been found to play a key role in fibrosis [12,13]. So far, HIPK2 has been found to be involved mainly in kidney fibrosis. However, a few studies have also started to show HIPK2 involvement in idiopathic pulmonary fibrosis (IPF), in cardiac and in liver fibrosis. For these reasons, HIPK2 is becoming an appealing molecule, both as biomarker and as therapeutic target in several different diseases due to its role as a central hub of a molecular network controlling several signaling pathways involved in cell death and proliferation, development and fibrosis.

## 2. Cellular and Molecular Mechanisms of Fibrosis

**Figure 2:**
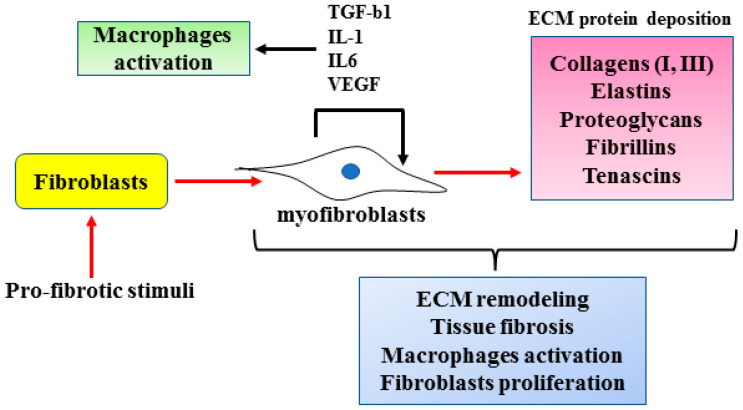
Cellular mechanisms regulating fibrosis. Under pro-fibrotic stimuli, fibroblasts, among the several cell types, as explained in the text, differentiate into myofibroblasts that produce extracellular matrix (ECM) components leading to tissue fibrosis. Myofibroblasts cross-talk with macrophages and this interplay contributes to tissue fibrosis.

At the cellular level the main cell types involved in fibrosis are fibroblasts and macrophages. Physiologically, the activation of fibroblasts is required for tissue remodeling such as wound healing after a cell injury. Fibroblasts become activated into myofibroblasts by inflammatory cytokines. Myofibroblasts display up-regulated cellular migration, exaggerated ECM production and increased chemical signaling secretion and responsiveness [5,14]. Myofibroblasts can arise not only from fibroblasts (Figure 2) but also from a variety of different cell types such as pericytes, endothelial cells, smooth muscle cells, epithelial cells, bone-marrow-derived fibrocytes and bone-marrow-derived progenitor cells [14,15]. Differentiation into myofibroblasts may initially be dependent on pro-fibrotic cytokines (e.g., TGFβ1, IL-1β, IL-1, IL-6, TNF*α*, etc.). However, when the regulatory mechanisms are disrupted, fibroblasts escape the normal control that is necessary for their inactivation, as soon as the damage is repaired, and may remain persistently activated [16]. As a result, the progressive deposition of ECM proteins (e.g., collagens, elastins, proteoglycan, fibrillins, tenascins, etc.) increases the stiffness of the affected organ, further promoting cell injury and myofibroblast activation [15,16]. The outcome of deregulated fibroblasts is tissue fibrosis which induces dysfunction or even loss of function of the affected organ [3]. Fibroblasts also participate in cancer progression by interacting with cancer cells. In response to that interaction, cancer-associated fibroblasts (CAF) secrete cytokines and ECM that modulate tumor cell growth, survival and migration by stimulating angiogenesis, hypoxia and escape to anti-tumor immunity; thus, tumors are often described as “fibrotic wounds that do not heal” [17]. In addition, fibroblasts produce and secrete cytokines such as TGFβ1, IL-1, IL-6, IL-33, VEGF, CXC and CC chemokines as well as reactive oxygen species (ROS) that induce macrophage activation [5,15]. The cellular mechanisms inducing myofibroblast activation leading to fibrosis are summarized in Figure 2.

Following tissue injury, an inflammatory response is activated and cytokines such as interferon (IFN)γ or tumor necrosis factor (TNF)α target macrophages that undergo marked phenotypic and functional changes to play a key role in all phases of tissue repair. Macrophages produce growth factors that promote cellular proliferation and blood vessel development [18]. When the healing process is under control, the inflammatory process resolves quickly and normal tissue architecture is restored. During prolonged tissue injury and a chronic inflammation, interleukin (IL)-4, IL-6 or IL-13 targets macrophages to produce pro-fibrotic mediators that facilitate the recruitment of inflammatory cells and promote the differentiation and activation of myofibroblasts, thus inducing fibrosis [18].

At a molecular level, the central mediator of fibrogenesis is TGFβ, a pluripotent growth factor that inhibits ECM degradation and promotes the expression of pro-fibrotic genes in several cell types [6,19]. TGFβ1 binds to type 2 TGF-beta receptor, allowing its dimerization with type 1 TGFβ receptor which leads to the phosphorylation of Smad2 and Smad3, with Smad2 being anti-fibrotic and Smad3 pro-fibrotic. Phosphorylated Smad3 translocates into the nucleus and activates the transcription of fibrotic genes such as collagen I, fibronectin and α-smooth muscle actin (αSMA) [20]. In this regard, the inhibition of TGFβ signaling exerts potent anti-fibrotic effects, as seen for instance in pulmonary fibrosis and in systemic sclerosis [20,21]; however, since TGFβ is also a negative regulator of the immune response by initiating T cell growth, its targeting has proven to be extremely difficult, if not impossible, in systemic treatment regimens [22,23].

Wnt/β-catenin is an evolutionarily conserved cellular signaling system that plays a crucial role in different biologic processes such as organogenesis and tissue homeostasis, as well as in the pathogenesis of many human diseases [24]. The key downstream effector in the Wnt/β-catenin pathway is β-catenin, a transcription factor that induces the expression of several target genes, including c-myc, cyclin D1 and vascular endothelial growth factor (VEGF), involved, respectively, in cell growth and angiogenesis [25]. The Wnt/β-catenin pathway may collaborate with TGF-β to control myofibroblast differentiation and promote matrix synthesis, playing an important role in fibrogenesis [26]. For that reason, the Wnt/β-catenin pathway may be considered a novel therapeutic target in fibrosis.

**Figure 3:**
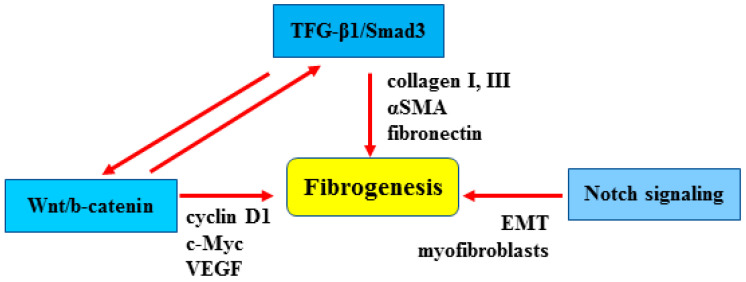
The Wnt/β-catenin, TGF-β1/Smad3 and Notch pathways contribute to fibrogenesis by stimulating myofibroblast differentiation and proliferation in order to produce extracellular matrix components.

Notch is an evolutionarily conserved intercellular signaling pathway that regulates interactions between physically adjacent cells and may also be involved in the development of fibrosis [8,27]. Activation of the Notch pathway leads to myofibroblast formation and to the phenotypic epithelial–mesenchymal transition (EMT) [28], while its inhibition has been proven to prevent the development of fibrosis in different models [8]. The involvement of the above-described molecular pathways in fibrosis is summarized in Figure 3. Inhibitors of TGF-β, Wnt, and Notch signaling pathways have not yet been tested or proven successful to target fibrosis in clinical trials [29]. Therefore, a deeper understanding of the molecular mechanisms underlying fibrosis can potentially be useful for identifying new biomarkers in order to develop novel and more efficient targeted therapies against fibrosis.

## 3. HIPK2 Function

**Figure 4:**
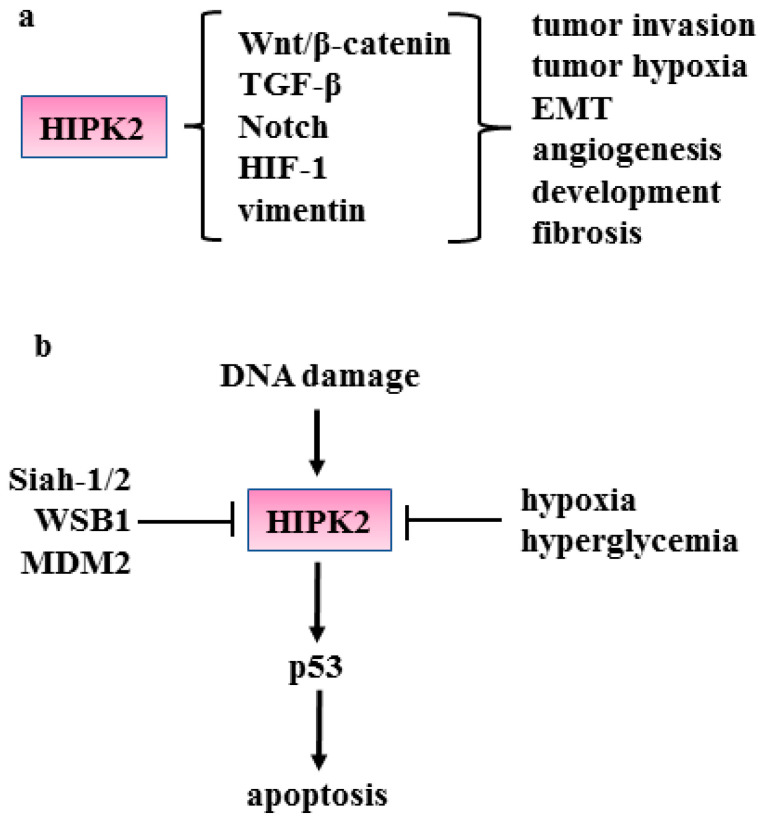
(**a**) Schematic representation of the molecular pathways regulated by HIPK2 and the underlying outcome. (**b**) HIPK2 is activated by DNA damage and consequently activates the p53 apoptotic function. HIPK2 is inhibited by hypoxia and hyperglycemia and by the Siah-1/2, WSB1 and MDM2 ubiquitin ligases. Black arrows indicate activation.

Homeodomain-interacting protein kinase (HIPK)2 is a nuclear serine–threonine kinase member of the HIPK family (numbered 1–4) that was originally identified to interact with homeodomain transcription factors (for a review see refs. [11,30,31,32,33,34]). HIPK2 protein is generally expressed at a low level due to its degradation by means of several ubiquitin ligases (e.g., seven in absentia homolog 1/2 (SIAH-1/2), WSB-1, and mouse double minute (MDM)2) [31] (Figure 4b). When activated, HIPK2 phosphorylates transcription factors and accessory components of the transcriptional machinery regulating the gene expression of several molecules involved in cell development, proliferation, apoptosis, and DNA damage response [32]. Depending on the cell context, HIPK2 can repress as well as promote transcription [33]. HIPK2 activity has been mainly studied in the cancer biology field. Here, we will briefly summarize the role of HIPK2 in cancer before describing its involvement in fibrosis in detail.

One of the best characterized functions of HIPK2 is the phosphorylation of p53 in serine 46 that specifically induces apoptosis-restraining tumor growth (Figure 4b). HIPK2 inhibition thus impairs the apoptotic function of p53 [34] and also induces a p53 mutant-like conformation that harms p53 oncosuppressor functions [35,36]. HIPK2 can regulate molecular pathways such as Wnt/β-catenin, TGF-β and Notch (Figure 4a) that are involved in fibrosis. HIPK2 has been found to suppress β-catenin-induced cyclin D1 transcription and restrict cell growth in a skin carcinogenesis model [37]. HIPK2 can phosphorylate β-catenin for proteasomal degradation [38] and such degradation leads to the transcriptional impairment of several β-catenin target genes, including the vascular endothelial growth factor (VEGF) involved in tumor angiogenesis and tumor growth [39]. In a model of Drosophila canonical Wg signaling during wing development, HIPK2 has been found to promote the stabilization of Arm/β-catenin in cell culture and in vivo [40] as well as to promote Notch signaling [41]. In breast cancer cells, HIPK2 phosphorylates and inhibits Notch1, thus impairing tumorigenesis [42]. HIPK2 also mediates the TGF-β-induced apoptosis in human hepatoma cells [43] and is required for the TGF-β-mediated survival of mouse neurons [44]. On the other hand, TGF-β signaling phosphorylates and activates HIPK2 to control angiogenesis [45]. HIPK2 can restrain tumor metastasis by downregulating vimentin, a driver for EMT and tumor invasion [46,47]. HIPK2 reduces hypoxia inducible factor 1 (HIF-1) activity (Figure 4a) by inhibiting the expression of the HIF-1α component of HIF-1 transcription factor [48]. HIPK2 inhibition has been shown to increase tumor progression and resistance to therapies by, for instance, activation of the β4 integrin signaling pathway and of the HIF-1-mediated cyclooxygenase-2 (COX-2)/prostaglandin E2 (PGE2) axis [49,50,51]. HIPK2 inhibition leads to p53 disfunction, responsible not only for cancer progression but also Alzheimer’s Disease (AD) pathogenesis [52]. The HIPK2 gene rarely mutates while HIPK2 protein can be inhibited by mechanisms activated by hypoxia or hyperglycemia [48,53] (Figure 4b), two conditions involved in tumor progression and also in fibrosis [54,55,56,57].

These findings highlight the critical role for HIPK2 in tumor growth and invasion but also in development and neurodegenerative diseases (for a more detailed summary, please refer to refs. [11,30,31,32,33,34]). The specific involvement of HIPK2 in fibrosis will be summarized in the paragraphs below.

### 3.1. HIPK2 in Renal Fibrosis

**Figure 5:**
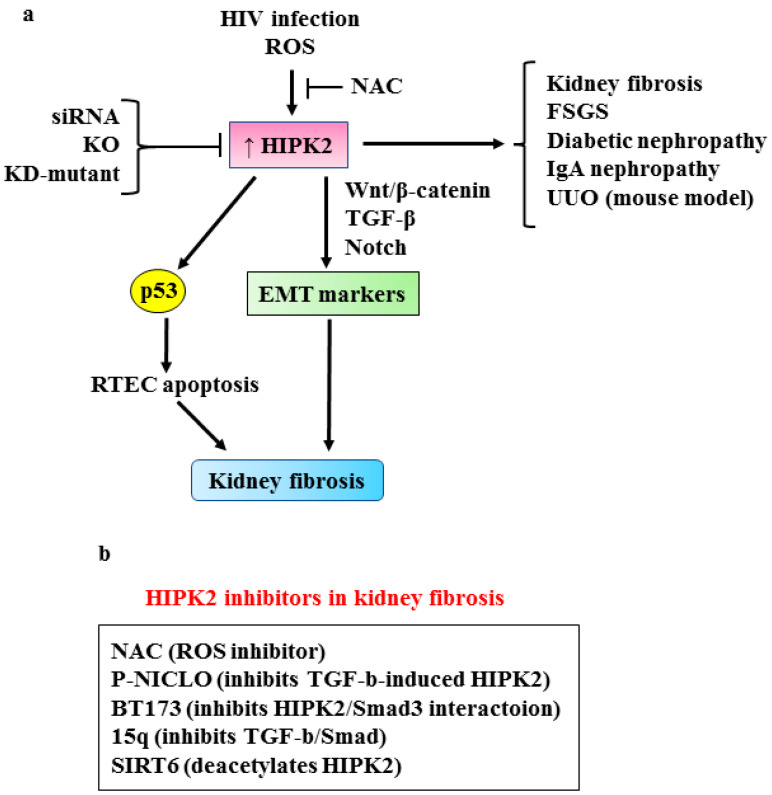
(**a**) HIPK2 role in kidney fibrosis. Upstream factors (HIV infection, oxidative stress-ROS) induce HIPK2 expression that consequently activates p53-dependent RTEC (renal tubular epithelial cells) apoptosis and the EMT (epithelial mesenchymal transition) markers by the pro-fibrotic pathways (Wnt/β-catenin, TGF-β and Notch), leading to kidney fibrosis. HIPK2 is overexpressed in kidney fibrosis, FSGS (focal segmental glomerulosclerosis), diabetic nephropathy, IgA nephropathy (IgAN) and unilateral urethral obstruction (UUO). NAC (N-acetylcysteine) inhibits ROS-induced HIPK2 upregulation. Black arrows indicate activation. (**b**) Schematic representation of HIPK2 inhibitors in renal fibrosis.

Renal fibrosis is the final step of chronic kidney diseases (CKD) characterized by the abnormal accumulation of extracellular matrix that eventually leads to end-stage renal disease (ESRD) [58,59], a major public health problem worldwide. Renal fibrosis is the consequence of the activation of several profibrotic pathways including TGF-β, Wnt/β-catenin and Notch [7,60,61,62,63,64,65]. In response to renal injury, tubular epithelial cells may undergo epithelial-to-mesenchymal transition (EMT) and express αSMA, becoming the mediators of the pathological fibrotic accumulation of ECM in CKD [66]. Recently, HIPK2 has been identified as a key regulator of inflammation and renal fibrosis [67]. Using an integrated computational and experimental systems biology approach, which takes into account protein–protein and protein–DNA interactions, the authors found HIPK2 to be a common regulator of signaling pathways activated in a mouse model of human immunodeficiency virus (HIV)-associated nephropathy (HIVAN) that presents both tubulointerstitial fibrosis and glomerulosclerosis [67]. The HIPK2 protein level increased in the kidneys of HIV transgenic mice (Tg26 mice) [68] as well as in those of patients with various kidney diseases [67]. Mechanistically, HIV infection increases HIPK2 protein stability by promoting oxidative stress which inhibits the seven in absentia homolog 1 (SIAH-1)-mediated HIPK2 proteasomal degradation [69].Thus, the inhibition of reactive oxygen species (ROS) by N-acetylcysteine (NAC) abrogates the HIV-induced HIPK2 upregulation and reduces fibrosis, confirming the positive role of HIPK2 in kidney fibrosis [67]. The increased HIPK2 stability is also responsible for the p53-induced apoptosis of renal tubular epithelial cells (RTECs) as well as for the expression of EMT markers, including αSMA, collagen I and fibronectin, in kidney epithelial cells by activating the TGF-β–Smad3, and Wnt-Notch pathways [67] (Figure 5a). As a proof-of-principle, the abrogation of HIPK2 activity by several means (silencing with small interfering (si)-RNA, overexpression of kinase-defective (KD)-mutant, HIPK2-knock-out (KO)) attenuates kidney fibrosis in Tg26 mice, as well as in other murine models of kidney fibrosis, such as unilateral urethral obstruction (UUO) and folic-acid-induced renal fibrosis, improving renal function and reducing proteinuria [67]. Interestingly, HIPK2’s role was confirmed in focal segmental glomerulosclerosis (FSGS), diabetic nephropathy and IgA nephropathy (IgAN), with the latter being the most prevalent primary human glomerulonephritis. These findings support a more general role for HIPK2 in human kidney diseases, highlighting its targeting as a potential new strategy in anti-fibrosis therapy [70] (Figure 5a). In this regard, it has been shown that phosphate niclosamide (P-NICLO), the water-soluble form of NICLO which is a US Food and Drug Administration-approved oral anti-helmintic drug [71], significantly inhibits the progression of renal fibrosis in a UUO mouse model [72]. The effect of P-NICLO is partly dependent on the inhibition of TGF-β-induced HIPK2 expression that consequently blocks the downstream pathways involved in fibrosis, including Smad, Notch, NF-kB and Wnt/β-catenin [72]. These findings demonstrate for the first time the anti-fibrotic effect of P-NICLO in renal fibrosis in mouse models.

Subsequently, by analyzing several compounds using the structure–activity relationship (SAR) approach, BT173 compound was found to be the most effective HIPK2 inhibitor, particularly in reducing the interaction between HIPK2 and Smad3, thereby inhibiting Smad3 phosphorylation and activation [73]. It is also of note that BT173 does not inhibit HIPK2 kinase activity or p53 activation, protecting in this way the tumor suppression functions regulated by HIPK2 [73]. On the other hand, as a consequence of the inhibition of the TGF-β1/Smad3 pathway, the in vivo administration of BT173 decreases renal fibrosis in UUO and Tg26 mouse models. The inhibition of the Wnt/β-catenin pathway by BT173, directly through HIPK2 inhibition or indirectly through TGF-β pathway inhibition, further enhances its anti-fibrosis effect [73].

Recently, other molecules have been synthesized as HIPK2 inhibitors in renal fibrosis. Among several compounds containing benzimidazole and pyrimidine scaffolds, compound 15q was found to have a potent inhibitory activity against HIPK2 and, as a proof of principle, it was tested on renal cells and on mouse models of renal fibrosis [74]. In vitro, in rat renal fibroblast (NRK-49F) cells, 15q inhibits HIPK2 activity and its downstream pro-fibrotic pathways, such as TGF-β1/Smad3, showing significant anti-fibrotic effect. In vivo, in UUO mouse models, 15q displays a potent anti-fibrotic effect. Interestingly the 15q-induced HIPK2 inhibition does not affect HIPK2 anti-tumor activity, underscoring selective HIPK2 targeting as an anti-fibrosis drug [74]. The HIPK2 inhibitors have been summarized in Figure 5b.

HIPK2 induction during fibrogenesis was proven to be blocked by Sirtuin 6 (SIRT6) overexpression [75] (Figure 5b). SIRT6 belongs to the conserved nicotinamide adenine dinucleotide (NAD+)-dependent chromatin deacetylase family, implicated in genomic stability, inflammation and energy metabolism regulation [76]. Overexpressed SIRT6 protects against kidney fibrosis by epigenetically blocking β-catenin activity and target gene expression. [77,78]. SIRT6 deficiency significantly activates TGF-β signaling and organ fibrosis [79] and the knockdown of SIRT6 aggravates unilateral ureteral obstruction (UUO)-induced renal fibrosis [80]. In a model of SIRT6-transgenic (SIRT6-Tg) mice, the authors showed that the upregulation of HIPK2 in fibrotic kidney and in tubular epithelial cells undergoing EMT is blocked by SIRT6 overexpression, with the consequent inhibition of renal interstitial fibrosis and of renal function deterioration in CKD [75]. Mechanistically, SIRT6 inhibits HIPK2 at protein level [75], likely by deacetylation that has been proven to induce HIPK2 proteasomal degradation [81,82]. However, whether HIPK2 is directly deacetylated by SIRT6 remains to be elucidated. Although the protective role of SIRT6 in CKD is prospective, a SIRT6 drug target is still unavailable in clinical applications. In this regard, a population study indicated that elderly people with a high dietary intake of vitamin B3, the most used form of NAD precursors, have a lower risk of renal function decline [75]. This finding underscores the SIRT6/HIPK2 axis as a promising pharmacologic target in renal interstitial fibrosis in CKD. Therefore, the suppression of HIPK2 may potentially be a potential therapeutic approach against kidney fibrosis progression.

In a mouse model of vancomycin (VAN)-induced transition of acute kidney injury (AKI) to chronic kidney disease (CKD), HIPK2 overexpression, mediated by the epidermal growth factor receptor (EGFR)/signal transducer and activator of transcription (STAT)3 pathway, was identified as a key regulatory mechanism [83]. VAN-induced nephrotoxicity has been associated with renal cell apoptosis [84]. The authors found that HIPK2 expression levels are increased after VAN treatment, both in vitro and in vivo. The genetic or pharmacologic inhibition of HIPK2 attenuates the VAN-induced progression of AKI to CKD and prevents renal fibrosis, as evidenced by improved renal function, reduced tubular damage, and attenuated cell apoptosis [83]. Furthermore, the inhibition of STAT3 significantly suppresses HIPK2 overexpression, suggesting that STAT3 is correlated with HIPK2 expression. Thus, it was found that STAT3 can physically interact with the promoter region of HIPK2 [83]. HIPK2 can induce p53 apoptotic activation [34] and, intriguingly, p53 has been found to activate miR-192-5p and mediate VAN-induced AKI [85]. The role of p53 has been found in the fibrosis of several different tissues, including liver, kidney, lung and heart [86,87,88]. Therefore, we can speculate that p53 may be activated by HIPK2 in the VAN-induced AKI, becoming the HIPK2/p53 pathway that is a potential target for developing prevention strategies for renal fibrosis.

Finally, in an in vitro model of human kidney (HK)-2 cells, the authors found that the overexpression of miR-141 inhibits TGF-β1-induced EMT through the repression of HIPK2 via direct interaction with the 3’-untranslated region of HIPK2 [89]. The authors suggest that miR-141 may represent novel biomarkers and therapeutic targets in the treatment of renal fibrosis.

### 3.2. HIPK2 in Pulmonary Fibrosis

Fibrotic pulmonary pathologies include a group of serious and heterogeneous lung diseases such as pulmonary hypertension, chronic obstructive pulmonary disease (COPD), hypoxic/hyperoxic injury, mechanical injury, idiopathic pulmonary fibrosis (IPF), etc., which affect the respiratory system. Pulmonary fibrosis is mainly caused by the abnormal activation of alveolar macrophages and pulmonary fibroblasts. Pulmonary fibrosis scars and thickens lung tissues that do not expand as well as they should, making it hard to breath [90]. IPF is a chronic, age-related, progressive fibrotic disease of unknown etiology characterized by anatomical disruption of the lung due to fibroblast proliferation and extracellular matrix deposition, leading to lung dysfunction and eventually to death [91]. The mechanism of IPF remains elusive; however, it is well accepted that, in a genetically predisposed alveolar epithelium, an abnormal reparative response to recurrent micro damages induces the formation of fibroblast and myofibroblast foci that produce excessive collagen deposition [90]. Several treatments can help reduce the rate at which IPF gets worse, but there is currently no treatment that can stop or reverse the scarring of the lungs, making lung transplantation the only approach to improve patient survival [92].

The involvement of HIPK2 in pulmonary fibrosis has reported so far in few studies. In the fibrotic area of a mouse model of bleomycin-induced pulmonary fibrosis, HIPK2 expression was found to be downregulated [93]. The mouse model of pulmonary fibrosis is characterized by an increased number of collagen fibers in alveolar spaces and by severe damage of the lung alveolar structure, since bleomycin induces β-catenin and mesenchymal cell makers (αSMA, collagen I and collagen III) [93]. The authors found that lentiviral overexpression of HIPK2 counteracts the effect of bleomycin, reduces mouse lung fibroblast (MLF) proliferation and migration, and induces their apoptosis, suppressing pulmonary fibrosis [93]. On the other hand, HIPK2 downregulation enhances MLF proliferation and migration, protecting them from apoptosis. The authors show that treating fibroblasts with the Wnt/β-catenin pathway inhibitor XAV939 reverses the fibroblast activation induced by HIPK2 downregulation [93]. These findings confirm the key role of HIPK2 in MLF activation via the Wnt/β-catenin signaling pathway. β-catenin is often activated in pulmonary fibrosis and promotes lung fibroblast migration and proliferation [94], therefore its targeting is a promising strategy to counteract lung fibrosis. Blockading the Wnt/β-catenin pathway has been shown to attenuate bleomycin-induced pulmonary fibrosis [95] and to suppress the myofibroblast differentiation of lung resident mesenchymal stem cells [96]. In agreement with these findings, a previous study showed reduction in HIPK2 expression, due to a loss of heterozygosity (LOH) at HIPK2 locus 7q32.34, in human lung fibroblasts derived from IPF patients [97], although the molecular mechanisms underlying HIPK2 reduction and its correlation with IPF was not determined.

Interestingly, circular (circ)HIPK2 expression was found to be increased in fibroblasts of a model of silica-induced pulmonary fibrosis [98]. Biological assays and pharmacological techniques, combined with functional experiments, show that circHIPK2 and micro RNA (miR)-506-3p are likely involved in the silica-induced endoplasmic reticulum (ER) stress, through the expression of sigma-1 receptor (σ-1R). ER stress is then responsible for the migration and proliferation of human pulmonary fibroblasts, inducing the development of silicosis [98]. Circular RNAs are a novel class of naturally occurring non-coding (nc) RNAs and are known to play a role as miRNA sponges and to interact with RNA-associated proteins to regulate gene transcription [99]. Circular RNA are involved in the pathogenesis of many diseases including cancer and fibrosis [100,101] and, given their abundance and stability in body fluids, they can become useful biomarkers. In this regard, circRNAs from the HIPK2 gene have been detected as the most abundant circRNAs [98,102], making them a potential biomarker for tissue fibrosis progression.

Recently, a genetic variant of HIPK2 has been found to be significantly related to radiation pneumonitis in lung cancer patients treated with radiation therapy [103], underscoring the role for HIPK2 in tissue inflammation and fibrosis after irradiation.

### 3.3. HIPK2 in Cardiac Fibrosis

**Figure 6:**
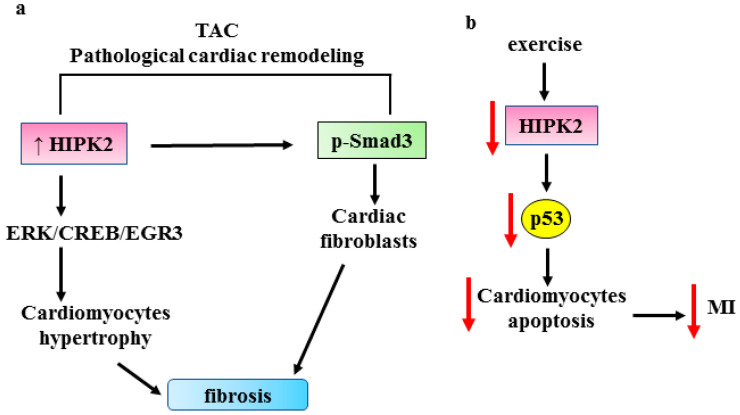
(**a**) Role for HIPK2 in cardiac fibrosis in a transverse aortic constriction (TAC) cardiac fibrosis model. The molecular pathways activated by HIPK2 are shown and they induce both cardiomyocyte hypertrophy and cardiac fibroblast proliferation, inducing cardiac fibrosis. (**b**) Schematic representation showing how exercise reduces myocardial infarction (MI) by downregulating HIPK2 with a consequent reduction in p53-induced cardiomyocyte apoptosis. Black arrows indicate activation. Red arrows indicate reduction/inhibition.

Heart failure is the final stage of many cardiovascular diseases and is one of the leading causes of death worldwide. It has poor prognosis as it lacks treatable clinical intervention targets. A risk factor for heart failure, following, for instance, hypertension or myocardial infarction, is pathological cardiac remodeling [104]. Recent studies highlighted a role for HIPK2 in cardiac remodeling. In an in vivo model of transverse aortic constriction (TAC), HIPK2 expression has been found to be elevated in cardiomyocytes. Mechanistically, the authors found that HIPK2 induces cardiomyocyte hypertrophy through the extracellular signal-regulated kinase (ERK)1/cyclic adenosine 3′,5′-phosphate (cAMP)-responsive element-binding protein (CREB) signaling pathway that regulates the early growth response 3 (EGR3) and C-type lectin receptor 4D (CLEC4D) targets [105] (Figure 6a). The genetic or pharmacologic inhibition of HIPK2 suppresses the cardiac fibrosis induced by TAC [105]. This finding is in agreement with the pro-fibrotic effect of HIPK2 seen in renal fibrosis [12,13]. The authors found that HIPK2 inhibition suppresses ERK1/CREB signaling and reverses stress-induced cardiomyocyte hypertrophy, a finding recapitulated in HIPK2^-/-^ mice; in addition, HIPK2 inhibition reduces Smad3 phosphorylation in cardiac fibroblast [105]. The latter finding is in agreement with a study showing that cardiomyocyte-specific Smad3 knockdown (KO) attenuates cardiac remodeling and infarction in mice [106], and with a study showing that HIPK2 knockdown attenuates Angiotensin (Ang) II-induced cardiac fibrosis, likely as a consequence of Smad3 inhibition [107]. The results suggest a potential role for HIPK2 as anti-fibrosis target in cardiac fibrosis. The findings by Zhou et al. suggest a potential role for HIPK2 as a target for the prevention of pathological cardiac remodeling and heart failure, in both cardiomyocytes and cardiac fibroblasts, although the protective effect of HIPK2 knockdown was only found in stressed conditions [105]. The same authors reported that exercise downregulates HIPK2, and that HIPK2 inhibition by lentiviral vectors protects from myocardial infarction attenuating cardiomyocyte apoptosis induced by oxygen glucose deprivation/reperfusion (OGD/R) [108]. Mechanistically, they found that p53 activator reverses the protective effect of HIPK2 inhibition against apoptosis [108] (Figure 6b), underscoring the apoptotic role of the HIPK2/p53 pathway in this setting. They also found that miR122, which targets HIPK2 [109], protects against cardiac dysfunction after myocardial infarction and that miR-122 serum levels are reduced in myocardial infarction patients. The authors suggest that miR122 could be a biomarker for the prognosis of myocardial infarction [108].

HIPK2 expression was found to be increased in cardiomyocytes exposed to hypoxia/reoxygenation (H/R), undergoing oxidative damage and apoptosis [110]. Intriguingly, HIPK2 overexpression was found to relieve H/R-induced cardiomyocyte apoptosis and oxidative damage [110]. Looking for the anti-apoptotic mechanism induced by HIPK2, the authors found that HIPK2 overexpression increases NRF2 antioxidant transcriptional activity [110] that has been shown to protect cardiomyocytes from H/R injury [111]. NRF2 inhibition partially reverses the HIPK2-mediated protective effect against H/R-induced cardiomyocyte oxidative damage and apoptosis [110]. The interplay between NRF2 and HIPK2 has been addressed in the past few years and it is yet not completely understood. We have shown that NRF2 activation inhibits the apoptotic HIPK2/p53 axis in cancer cells by promoting HIPK2 protein degradation, [53,112]. On the other hand, in some circumstances, NRF2 may induce HIPK2 gene transcription and direct HIPK2 transcriptional activity toward antioxidant genes that are in common with NRF2. In this manner, NRF2 and HIPK2 engage a pro-survival crosstalk to the detriment of HIPK2 apoptotic activity [113]. However, how HIPK2 activity can switch from apoptotic to pro-survival in the presence of NRF2 is still under debate and needs further clarification. Therefore, a better understanding of the mechanistic interplay between NRF2 and HIPK2 could help to elucidate not only the pro-survival/apoptotic outcome in cancer, especially in response to therapeutic agents, but also the induction of fibrosis.

Alternatively, HIPK2 was found reduced in human end-stage ischemic cardiomyopathy [114]. The authors found that adenovirus-mediated HIPK2 overexpression downregulates heart failure markers, concluding that HIPK2 is a key molecule to maintain basal cardiac function [114]. The discrepancy between the effect of HIPK2 in the Guo et al. study, where HIPK2 is found to be reduced in human end-stage ischemic cardiomyopathy [114], and in the Zhou et al. study, where HIPK2 is overexpressed in cardiomyocytes of a TAC model [105], could depend on different molecular strategies for generating HIPK2^-/-^ mice, or by the fact that the protective effect of HIPK2 knockdown was only found in stressed conditions [105]. Although there is evidence of a role of HIPK2 in pathological cardiac conditions, additional studies are necessary to uncover the regulatory mechanisms that involve HIPK2 in cardiac fibrosis, in order to find appropriate therapeutic options.

### 3.4. HIPK2 in Liver Fibrosis

Liver fibrosis is a major cause of morbidity and mortality from hepatic diseases and is characterized by the excessive deposition of ECM that eventually leads to cirrhosis and even to hepatocellular carcinoma (HCC) [115,116]. During liver injury, hepatic stellate cells (HSCs) undergo activation into contractile myofibroblasts expressing αSMA, and their activation contributes to increased fibrogenesis [117]. Therefore, the inhibition of HSC activation is a potential strategy to counteract liver fibrosis. In this regard, it has been shown that HIPK2 expression is up-regulated in liver fibrotic tissues and in TGF-β1-treated HSCs [118]. The inhibition of HIPK2 reduces the expression of mesenchymal markers and Smad3 phosphorylation [115], suggesting the potential involvement of HIPK2 in liver fibrosis. Interestingly, in a mouse model of sepsis, HIPK2 overexpression has been shown to protect hepatocytic cells from injury by activating calpain-mediated autophagy [119]. The authors also showed that resveratrol, aspirin, vitamin E and ursolic acid, which are reported to protect the liver from injuries, increase the levels of HIPK2 by regulating its promoter [119]. These findings indicate that HIPK2 can be a promising therapeutic target in sepsis. These data also suggest that HIPK2, by protecting hepatocytic cells from injury, might also protect the liver from consequent fibrosis. In agreement, there is an interesting link between liver fibrosis, SIRT6 and HIPK2. SIRT6 is demonstrated to protect the liver from fibrosis induced by deregulated HSCs [120,121], and SIRT6 overexpression has been shown to inhibit HIPK2 at the protein level with a reduction in renal interstitial fibrosis and renal function deterioration in CKD [73]. Therefore, the interplay between SIRT6 and HIPK2 is worth studying further in fibrotic diseases.

### 3.5. HIPK2 in Other Fibrotic Disorders

Abnormal wound healing can induce the formation of hypertrophic scars and keloids which lead to dysfunction and deformity. The mechanism of keloid formation is still under debate and, in this regard, EMT has been shown to play a key role [122]. In a model of primary normal skin and keloid HIPK2, it has been found overexpressed in keloid-derived keratinocytes compared to normal keratinocytes [123]. HIPK2 inhibition by small-interfering (si) RNA abrogates the EMT markers and Smad3 phosphorylation, becoming a potential pharmacologic target for anti-keloid therapy.

HIPK2 was found to be involved in inflammatory response during skeletal muscle wound healing in an animal model of skeletal muscle contusion [124]. The repair mechanisms needs both inflammation and tissue repair orchestrated by an interplay between macrophages and fibroblasts. The authors found that HIPK2 is largely expressed in neutrophils, macrophages and myofibroblasts in the contused tissues [124], suggesting that it may be involved in regulating skeletal muscle regeneration in mice. The authors conclude that HIPK2 may be a target for the treatment of skeletal muscle injury [124].

Recently, it has been reported that HIPK2, Smad3 phosphorylation and p53 phosphorylation are increased in rabbit corneal keratinocytes cultured with 10% FBS (fetal bovine serum) which induces the differentiation of keratinocytes into myofibroblasts [125]. The inhibition of HIPK2 suppresses the FBS-induced differentiation of keratinocytes into myofibroblasts. Mechanistically, the authors found that HIPK2 is a direct target of miR-19a that is found enriched in the exosomes derived from the adipose-derived stem cells (ADSCs). Therefore, ADSC-derived miR-19a can suppress FBS-induced keratinocyte differentiation into myofibroblasts by inhibiting HIPK2 [125].

### 3.6. HIPK2 and Cancer-Associated Fibroblasts

Cancer-associated fibrosis may contribute to cancer progression and resistance to anticancer therapies as a consequence of the interplay between cancer cells and the surrounding components embedded in the tumor microenvironment (TME), such as fibroblasts, immune system, vasculature and ECM components [126]. Cancer cells contribute to a fibrotic TME phenotype by inducing chronic inflammation, altered immune infiltration and hypoxia, conditions known to drive the development of cancer-associated fibrosis and cancer metastasis [127,128]. Cancer cells, by producing growth factors and cytokines, recruit the TME fibroblasts and induce their differentiation into cancer-associated fibroblasts (CAFs) expressing α-SMA, which contribute to tumor progression [17]. A direct role of HIPK2 in cancer-associated fibrosis is still an unresolved issue, although its function in fibrosis is starting to be unveiled, as seen above. In an in vitro study, we have shown that the inhibition of HIPK2 in cancer cells induces oxidative stress with increased ROS generation that leads to autophagy-induced fibroblast differentiation into CAF [129]. We also found reduced p53 expression in fibroblasts undergoing CAF differentiation, although the mechanism is still unresolved [129]. Interestingly, the loss of p53 in stromal fibroblasts and in patient-derived CAFs has been shown to enhance tumor growth [130]. These findings are in favor of a potential role for HIPK2 in limiting cancer-associated fibrosis. Interestingly, HIPK2 inhibition can be achieved in cancer cells by tumor hypoxia or by hyperglycemia [48,53], two conditions involved in both tumor progression and in fibrosis [54,55,56,57]. Therefore, it is tempting to expand the role of HIPK2 in cancer by also adding the function that restrains cancer-associated fibrosis, although this hypothesis needs to be further clarified

## 4. Conclusions

**Figure 7:**
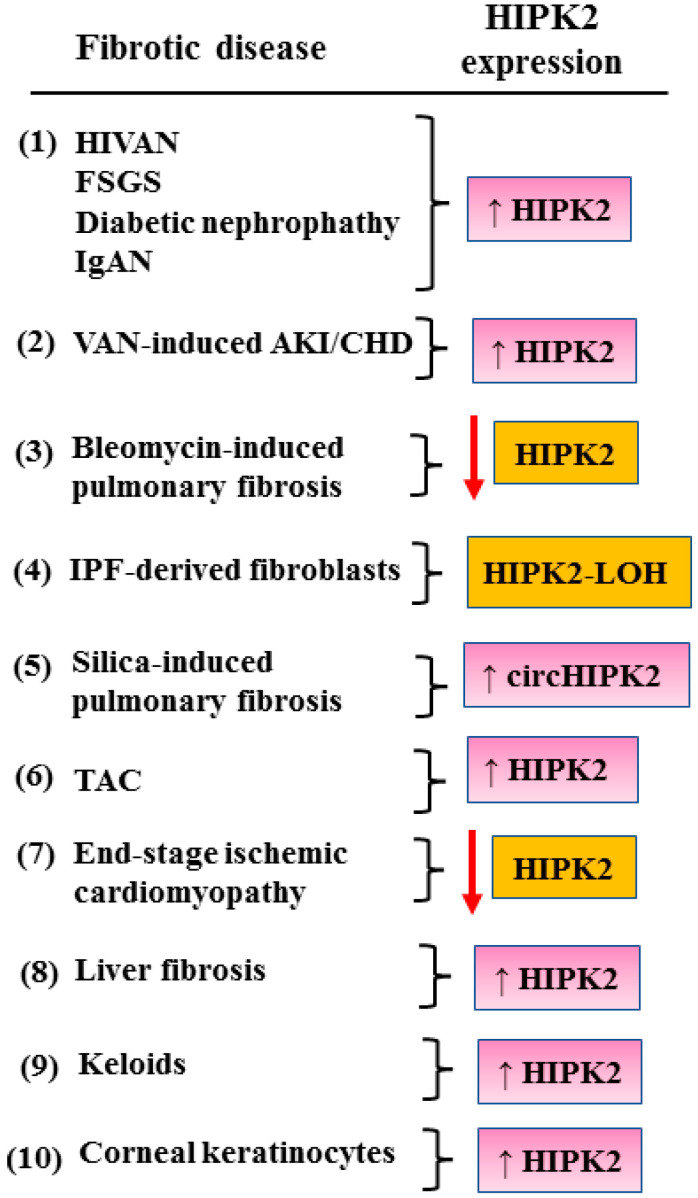
Summary of the HIPK2 expression in the several different fibrotic diseases discussed in the review. Black arrows indicate upregulation. Red arrows indicate downregulation. LOH = loss of heterozygosity.

Fibrotic disorders represent an increasing cause of morbidity and mortality worldwide. These diseases have a poor prognosis and are uncurable. Despite the effort in the research field of fibrosis, the absence of appropriate and fully validated biomarkers negatively impacts on the effectiveness of the therapeutic approaches. In this regard, HIPK2 is a promising biomarker for the progression of fibrosis in different tissues (Figure 7) and as a potential therapeutic target that is worth further study. However, given the oncosuppressing role of HIPK2, its manipulation needs to be performed with caution to avoid unwanted side effects.
